# Potassium levels after liver reperfusion in adult patients undergoing cadaveric liver transplantation: A retrospective cohort study

**DOI:** 10.1016/j.amsu.2020.05.002

**Published:** 2020-05-16

**Authors:** Laurence Weinberg, Dong-Kyu Lee, Anoop Ninan Koshy, Kai Wen Leong, Shervin Tosif, Ruth Shaylor, Param Pillai, Lachlan Fraser Miles, Alexandra Drucker, Brett Pearce

**Affiliations:** a Austin Health, Victoria, Australia; b Department of Surgery, Austin Health, University of Melbourne, Victoria, Australia; c Department of Anesthesiology and Pain Medicine, Korea University, Guro Hospital, Guro-Gu, Seoul, Republic of Korea; d Department of Cardiology, Austin Health, Victoria, Australia; e Department of Anesthesia, Austin Health, Victoria, Australia; f Intensive Care Unit, Austin Health, Victoria, Australia

**Keywords:** Liver transplantation, Potassium, Reperfusion, Anesthesia

## Abstract

**Background:**

Hyperkalemia is a common cause of arrhythmias in patients undergoing liver transplantation. We examined the pattern of change of potassium levels during and immediately after reperfusion of the donor liver.

**Materials and methods:**

Potassium levels of 30 consecutive adult patients undergoing cadaveric liver transplantation were assessed before and after liver reperfusion. Changes in potassium levels over 13 predefined timepoints were analyzed. Primary aim: to describe the pattern of change of potassium levels during the reperfusion period. Correlation between changes in potassium levels during reperfusion and a-priori variables were investigated.

**Results:**

Baseline median (IQR) potassium levels were 4.1 (3.8:4.5) mmol/L. Thirteen patients (43%) developed hyperkalemia, 10 (33%) of whom developed severe hyperkalemia. Potassium levels peaked at 80 s post reperfusion, plateaued until 2 min, before returning toward baseline values at 5 min. There was a strong association between pre-reperfusion/baseline potassium levels and peak potassium values during reperfusion (95%CI: 0.26 to 0.77, p < 0.001). A baseline potassium level of 4.45 mmol/L was a good predictor of reperfusion hyperkalemia with a sensitivity of 69.2% and specificity of 94.1% (AUC = 0.894, 95%CI: 0.779 to 1.000, p < 0.001).

**Conclusion:**

Hyperkalemia during cadaveric liver transplantation is common affecting almost 1 in 2 patients during reperfusion. During reperfusion potassium levels peaked within 2 min and over a third of patients developed severe hyperkalemia. Higher peak potassium levels correlated strongly with higher pre-reperfusion potassium values. These findings guide clinicians with timing of sampling of blood to check for hyperkalemia and identify modifiable factors associated with the development of hyperkalemia.

## Highlights

• Hyperkalemia during the reperfusion phase of cadaveric liver transplantation is common.
• Potassium levels peaked at 80 s post reperfusion, plateaued for 2 min, and returned to baseline at 5 min.
• There was a strong association between pre-reperfusion potassium levels and peak potassium values during reperfusion.
• Timing of blood sampling to check for hyperkalemia is paramount.
• A baseline potassium level of greater than 4.45 mmol/L was a good predictor of reperfusion hyperkalemia.

## 1 Introduction

Orthotopic liver transplantation (LT) is a complex surgical procedure associated with significant morbidity and a small mortality. A key anesthetic consideration during reperfusion of the donor liver is the vigilant monitoring and management of intraoperative potassium levels [1]. The occurrence of post reperfusion cardiac arrest is often unpredictable, with underlying etiologies including pulmonary embolism, anaphylaxis, intracardiac thrombus, and acute coronary events. In addition, severe hyperkalemia is a potentially modifiable risk factor associated with post reperfusion cardiac arrest and post reperfusion syndrome [2]. Severe hyperkalemia at reperfusion can result in cardiac arrhythmias and asystole if not adequately monitored and treated [[3], [4], [5], [6]]. The effects of elevated recipient potassium levels and subsequent severe hyperkalemia have been reported as being an independent risk factor for postoperative mortality [3].

Abrupt derangements in plasma potassium are particularly frequent during the reperfusion stage of LT [4,5], and both donor and recipient factors can contribute to the frequency and severity of changes in potassium levels. Clamping of the inferior vena cava, hepatic and portal veins during the anhepatic stage may result in the accumulation of potassium due to impaired renal function or large blood transfusions [4], and subsequent unclamping of these vessels at reperfusion results in the release of accumulated potassium as well as distribution of preservation fluid and other unidentified vasoactive substances from the donor liver into the recipient circulation [4,6].

Despite the growing recognition of the consequences of intraoperative hyperkalemia, there still appears to be a gap in knowledge of when potassium levels peak after liver reperfusion. Therefore, the primary aim of this observational study was to accurately describe the pattern of change of potassium levels during the immediate reperfusion period. Our secondary aims were to evaluate the number of patients who developed hyperkalemia and severe hyperkalemia after reperfusion, and to assess any correlation between changes in potassium levels during reperfusion and prespecified perioperative variables.

## 2 Material and methods

The study was conducted at a quaternary level university teaching hospital. To date over 1200 transplants have been undertaken by a multidisciplinary team of 5 transplant surgeons and 9 anesthesiologists. The study was approved as a quality improvement project by the Human Research Ethics Committee. There was no change to standard clinical care for any participant. The study was non-interventional and retrospective in design, and the need for informed written consent from participants was waived. The study was retrospectively registered with the Australian New Zealand Clinical Trials Registry (ANZCTR no:12619001488190).

We included consecutive adult patients (>18 years of age) undergoing primary cadaveric (donation after brain death) LT between July 2017–Jan 2018. We excluded patients undergoing donation after cardiac death transplantation, multiorgan transplantation, pregnancy, fulminant liver failure, end stage renal disease (serum creatinine >300 mmol/L), and patients requiring preoperative renal replacement therapy or intraoperative continuous venous-venous hemofiltration. As part of routine perioperative optimization, all patients underwent a comprehensive preoperative prehabilitation program that included optimization of hemoglobin according to the National Blood Authority of Australia's patient blood management initiative [7], glycemic optimization according to the Australian Diabetes Society guidelines [8], exercise rehabilitation and nutritional optimization according to our local hospital policies. In addition, anesthesia and surgical care were managed using a protocol designed to standardize patient care, as previously described [9]. Preoperative management of potassium was at the discretion of the treating medical transplant team and there were no individualized potassium management protocols specific to the transplant patients.

For all crystalloid fluid intervention during the LT we used Plasma-Lyte 148 solution (Baxter Healthcare, Toongabbie, NSW, Australia), a physiological non-lactated acetate buffered fluid. For colloid fluid intervention, we used 20% Albumin (Albumex-20™, CSL Behring Pty Ltd., Victoria, Australia). The physiochemical electrolyte composition of Plasma-Lyte 148 is similar to human plasma and includes potassium (5 mmol/L), sodium (140 mmol/L), chloride (98 mmol/L) magnesium (1.5 mmol/L), acetate (27 mmol/L), and gluconate (17 mmol/L). The physiochemical electrolyte composition of 20% Albumin includes albumin (20 g/L), sodium (40–100 mmol/L) and octanoate (32 mmol/L). All organs were preserved with Belzer UW® Cold Storage Solution (Preservation Solutions, Inc, Elkhorn, WI) cooled to 2° to 6 °C (36°–43°F). There was no warm machine perfusion used. The Belzer UW® Cold Storage Solution was left in the organ vasculature during hypothermic storage and transportation. The physiochemical components of the cold storage solution include sodium (29 mEq/L), a potassium (125 mEq/L), pH of approximately 7.4 at 20 °C (calculated osmolarity of 320 mOsm). The donor organ was flushed free of the Belzer UW® Cold Storage Solution with physiological solution (saline 0.9%) to prevent occurrence (in the recipient) of potentially serious cardiovascular complications such as hyperkalemic cardiac arrest or bradyarrhythmia. After completion of surgery, all patients were transferred to the intensive care unit (ICU) for ongoing monitoring and postoperative care and were weaned from mechanical ventilation according to standardized ICU protocols.

### 2.1 Standardization of blood gas sampling

To minimize preanalytical variability in the collection, handling and transportation of blood gas samples, a single skilled clinician performed all blood gas aspirates using a standard sampling protocol. Intraoperatively, as part of our standardized LT protocol, all patients had a right femoral arterial line inserted for continuous blood pressure monitoring and an additional 20 gauge left radial arterial line inserted to allow for frequent arterial blood sampling for the measurement of potassium levels, in addition to other standard physiological variables.

Prior to reperfusion, the donor liver was continually flushed with 2 L of saline 0.9% until the effluent fluid of the donor liver was less than 10 mmol/L. After reperfusion, arterial blood from each patient was sampled at the following 13 timepoints; i) immediately prior to reperfusion (baseline potassium level) ii) every 20 s after reperfusion for a duration of 3 min (9 samples), and iii) every 30 s until 5 min post-reperfusion (4 samples). During reperfusion a continuous electrocardiograph (ECG) was recorded to evaluate for ECG changes of hyperkalemia. Electrolyte abnormalities, including clinically significant hyperkalemia and reperfusion arrhythmias were managed as per standard anesthesia care.

Whole blood was sampled from the 20-gauge radial arterial line using a standardized sampling process. First, an 8 mL sample of arterial blood was slowly aspirated into an 8 mL discard syringe minimizing any force or strain during the aspiration process. This discard blood was returned to the cell saver to minimize blood wastage. Then, a further 1.0 mL of arterial blood was slowly aspirated from the radial arterial line over a 2 s period and placed into a safePICO™ blood gas aspirator (Radiometer Medical Aps, Brenshel, Denmark) containing 80 IU electrolyte balanced heparin. This blood sampling technique was used to minimize any in-vitro hemolysis, which can falsely increase values of plasma constituents, especially potassium. The safePICO™ aspirator has a clear 1.0 mL label designed for accurate blood sampling that allows an exact amount of blood necessary to produce reliable results; this further contributes to our intraoperative hemoglobin optimization program.

The blood sample syringe was transported horizontally and at standard room operating temperature (22 °C) to an automated blood gas analyzer (ABL800, Radiometer, Denmark) located in the same operating room. The same blood gas analyzer was used for all measurements. This blood gas platform utilizes an automated micromode to eliminate any risk of user-induced bias or loss of accuracy with very small samples. Further, the system uses an automatic mixing system integrated in the gas analyzer aiding in obtaining a homogenous sample for correct results and avoids vigorous manual mixing which can also lead to a hemolyzed blood gas sample [10]. As part of our institutions standard processes, the ABG analyzer is calibrated regularly to ensure results are concordant with the electrolyte values obtained by serum samples. The normal reference range for potassium on the ABG analyzer is 3.3–5.0 mmol/L.

### 2.2 Key study objectives

The key objective was to describe the pattern of change of potassium levels after liver reperfusion. The primary aim was to assess the time points when plasma potassium peaked after reperfusion. At each time point that blood was sampled after liver reperfusion, we evaluated the absolute potassium value at that time point in addition to changes in potassium values from baseline or pre-reperfusion values.

Our secondary aims were to evaluate the number of patients who developed hyperkalemia or severe hyperkalemia after reperfusion. Hyperkalemia was defined as a potassium level above 5.5 mmol/L and severe hyperkalemia as a potassium level greater or equal to 6.0 mmol/L [11]. We also assessed if there were any ECG features of hyperkalemia. A continuous ECG recording was performed during the reperfusion period. The ECG was evaluated for features of reperfusion hyperkalemia including peaked T waves, prolongation of PR interval, widening of the QRS complex, loss of P wave, “sine wave”, and asystole. The ECGs were reviewed by two independent clinicians, including a cardiologist, both of whom were blinded to the potassium values of each participant. Finally, we assessed any correlation between changes in potassium levels during reperfusion and the following a-priori variables: MELD (Models for End-stage Liver Disease) score, cold and warm ischemia times, vasopressor use at the time of reperfusion, pre-potassium effluent levels of the donor liver, and pre-reperfusion potassium levels.

### 2.3 Statistical methodology

Continuously distributed data was tested for normality and measures of central tendency were analyzed. Normally distributed data were expressed as means (standard deviations [SD]) and analyzed using parametric statistical methods including a Student's t-test; non-normally distributed data were expressed as medians (interquartile range [IQR]). Categorical variables were described as proportions and compared using the Chi-squared test or Fisher's Exact test. For the primary outcome evaluating the pattern of change of potassium over the pre-specified time points we used the Friedman test followed by Dunn's pairwise post hoc test. The strength of the relationship and association between the changes in potassium levels and the a-priori variables described above were evaluated with the Spearman rank correlation coefficient. To estimate the threshold of preoperative potassium level prone to reperfusion hyperkalemia, we introduced the Receiver-Operator Curve (ROC) analysis and defined the value based on the highest test sensitivity and specificity. The study has been reported in line with the Strengthening the Reporting Of Cohort Studies in Surgery (STROCCS) criteria [12].

## 3 Results

Thirty consecutive participants undergoing cadaveric liver transplantation were included. All participants received standard anesthetic management based on current liver transplantation anesthetic protocols described above. There were no violations or breaches in either the blood sampling of arterial blood or in the measurement technique for the assessment of potassium values.

### 3.1 Baseline characteristics

Of the thirty participants recruited, 20 (67%) patients were female, and 10 (33%) were male. The median (IQR) age and body mass index was 57.5 years (46:64.5) and 25.2 kg/m^2^ (22.9:27.5) respectively. MELD score ranged between 7 and 46 with a median (IRQ) of 21.0 (15.5:26.3). Common indications for transplant were viral hepatitis (23 [64%]) and alcoholic liver cirrhosis (10 [33%]). Some patients had more than one indication for transplant. The median (IQR) preoperative albumin was 33 g/L (30:39). The median (IQR) warm and cold ischemia times were 46.0 min (41.0:55.7) and 365.0 min (240.0:407.3) respectively. Preoperative patient characteristics are summarised in Table 1.

**Table 1:** Preoperative patient characteristics. Data presented as median (interquartile range) or number (proportion).

Variable	Result
Age (years)	57.5 (46.0:64.5)
Body Mass Index (kg/m^2^)	25.2 (22.9:27.5)
MELD score^a^	21.0 (15.5:26.3)
**Indication for transplant**^b^	
Viral Hepatitis	23 (64%)
Alcoholic cirrhosis	10 (33%)
Hepatoma	8 (27%)
Primary sclerosing cholangitis	5 (17%)
Non-alcoholic steatohepatitis	4 (13%)
Primary biliary cirrhosis	2 (7%)
Other^c^	6 (20%)
Hemoglobin (g/L)	93.5 (76.7:121.3)
White cell count (10^9^/L)	5.2 (3.5:6.9)
Platelets (10^9^/L)	74.0 (60.5:154.3)
Hematocrit (%)	30.0 (20.0:40.0)
International Normalised Ratio (IU)	1.7 (1.3:2.4)
Sodium (mmol/L)	137.0 (134.8:140.5)
Potassium (mmol/L)	4.0 (3.6:4.4)
Chloride (mmol/L)	100 (96.0:104.0)
Bicarbonate (mmol/L)	23.0 (20.5:26.0)
Urea (mmol/L)	7.3 (5.3:13.7)
Creatinine (*μ*mol/L)	91.0 (75.2:120.3)
Estimated Glomerular Filtration Rate (mL/min/1.73m^2^)	73.5 (53.0:88.5)
Calcium (mmol/L)	2.2 (2.1:2.3)
Magnesium (mmol/L)	0.8 (0.7:0.9)
Phosphate (mmol/L)	1.1 (0.9:1.2)
Bilirubin (*μ*mol)	96.0 (19.7:278.3)
Alanine transaminase (U/L)	35.0 (26.7:104.5)
Aspartate transaminase (U/L)	170 (131:266)
Gamma-glutamyl transferase (U/L)	62.0 (27.7:164.0)
Alkaline phosphatase (U/L)	119.0 (75.2:201.3)
Total protein (g/L)	66.0 (56.5:72.0)
Albumin (g/L)	33.0 (30.0:39.0)

a : Model for End-stage Liver Disease score.

b Some patients had more than 1 indication for transplantation.

c Intravenous drug use, secondary biliary cirrhosis, autoimmune lupoid, angiosarcoma, flucloxacillin induced vanishing bile duct syndrome, biliary malignancy.

### 3.2 Key outcomes

The median (IQR) potassium level immediately prior to reperfusion was 4.1 mmol/L (3.8:4.5). No patient was hyperkalemic at this time point. After reperfusion there were significant changes in potassium levels over the measured time points (Friedman test, Chi-squared value of 152.966, df = 12, p < 0.001). Thirteen patients (43%) developed hyperkalemia (potassium value > 5.5 mmol/L) during the reperfusion period. Of these patients, 10 (33%) developed severe hyperkalemia (potassium value > 5.9 mmol/L). One patient developed life-threatening hyperkalemia 40 s after reperfusion with a peak potassium of 8.0 mmol/L. Within 20 s, this value decreased to 6.1 mmol/L, and by 2 min the potassium level had normalised (below 5.0 mmol/L).

**Fig. 1:**
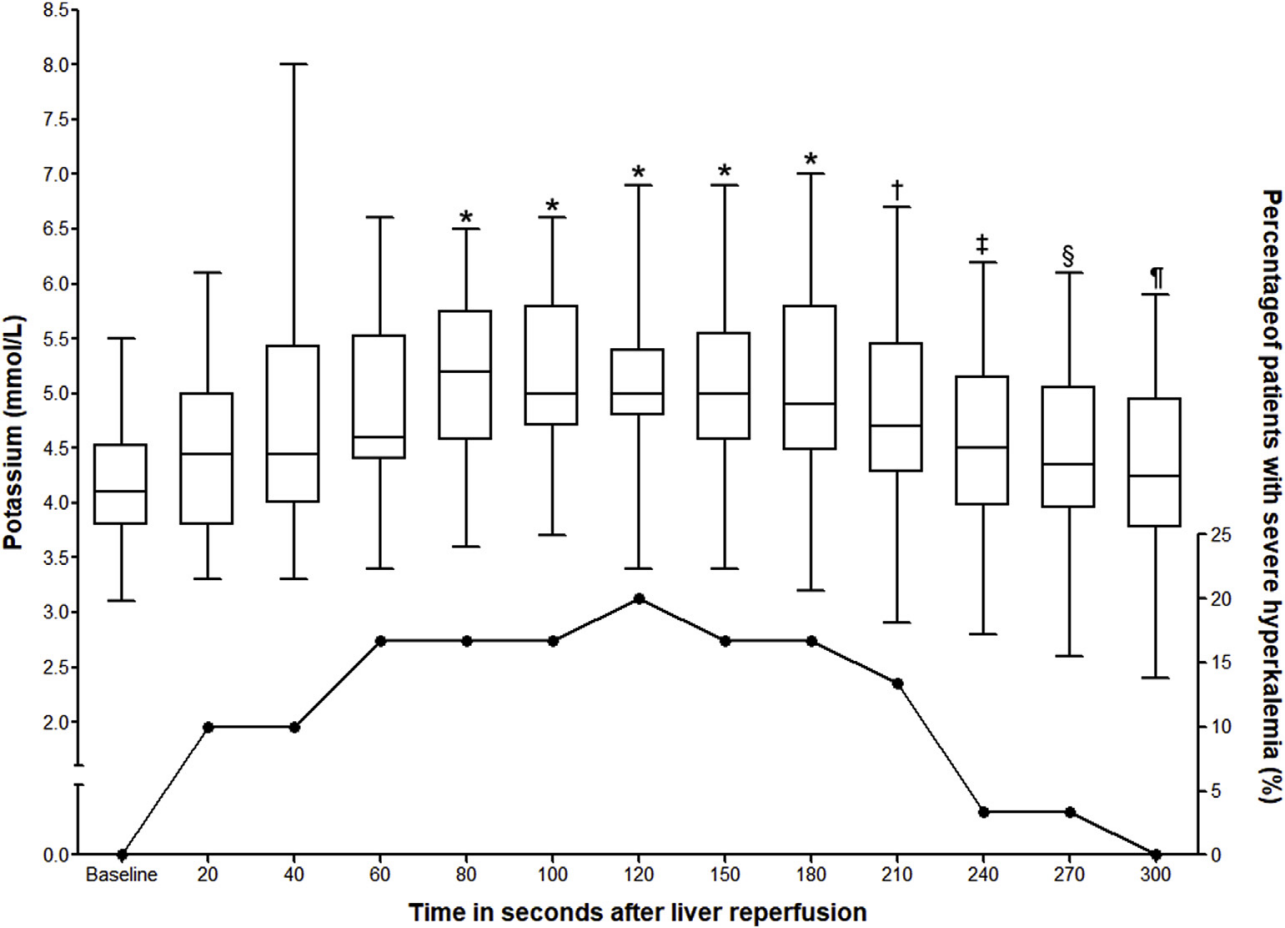
Potassium level changes and incidence of severe hyperkalemia at each time point. Box-Whisker plot represent the absolute potassium levels after liver reperfusion in patients undergoing deceased donor liver transplantation (Friedman test, Chi-squared value of 152.966, df = 12, p < 0.001). Line chart represents the incidence of severe hyperkalemia (potassium level greater than or equal to 6.0 mmol/L) at each time point after liver reperfusion. Severe hyperkalemia was increased around 120 s after reperfusion (Fisher-Freeman-Halton exact test, P = 0.026). *: p < 0.05 vs. Baseline, 20 and 40 s, †: p < 0.05 vs. Baseline only, ‡: P < 0.05 vs. 80, 100 and 120 s, §: p < 0.05 vs. 80, 100, 120, 150 and 180 s, ¶: p < 0.05 vs. 80, 100, 120, 150, 180 and 210 s.

Changes in potassium levels and the incidence of hyperkalemia are presented graphically in Fig. 1. The boxes represent the median, 25th and 75th centile values and the whiskers represent the minimum and maximum potassium levels at each time point. The line chart presents incidence of severe hyperkalemia (potassium level greater or equal to 6.0 mmol/L) at each time point after liver reperfusion. As presented by the box plots, the median potassium levels peaked at 80 s post reperfusion, plateaued for a further minute, before returning toward baseline values at 5 min. Baseline potassium levels were higher in patients who developed hyperkalemia during reperfusion compared to the patients without hyperkalemia and its effect size was large - 4.50 [0.49] mmol/L for the patients who experienced hyperkalemia, 3.74 [0.42] mmol/L for the patients without hyperkalemia, Student's t-test, mean difference = 0.76 mmol/L, 95%CI: 0.41 to 1.18, p < 0.001, Cohen's d = 1.64. The incidence of severe hyperkalemia (potassium level greater or equal to 6.0 mmol/L) at each time point after liver reperfusion is presented graphically by the line chart in Fig. 1. Severe hyperkalemia was increased around 120 s after reperfusion (Fisher-Freeman-Halton exact test, p = 0.026).

**Fig. 2:**
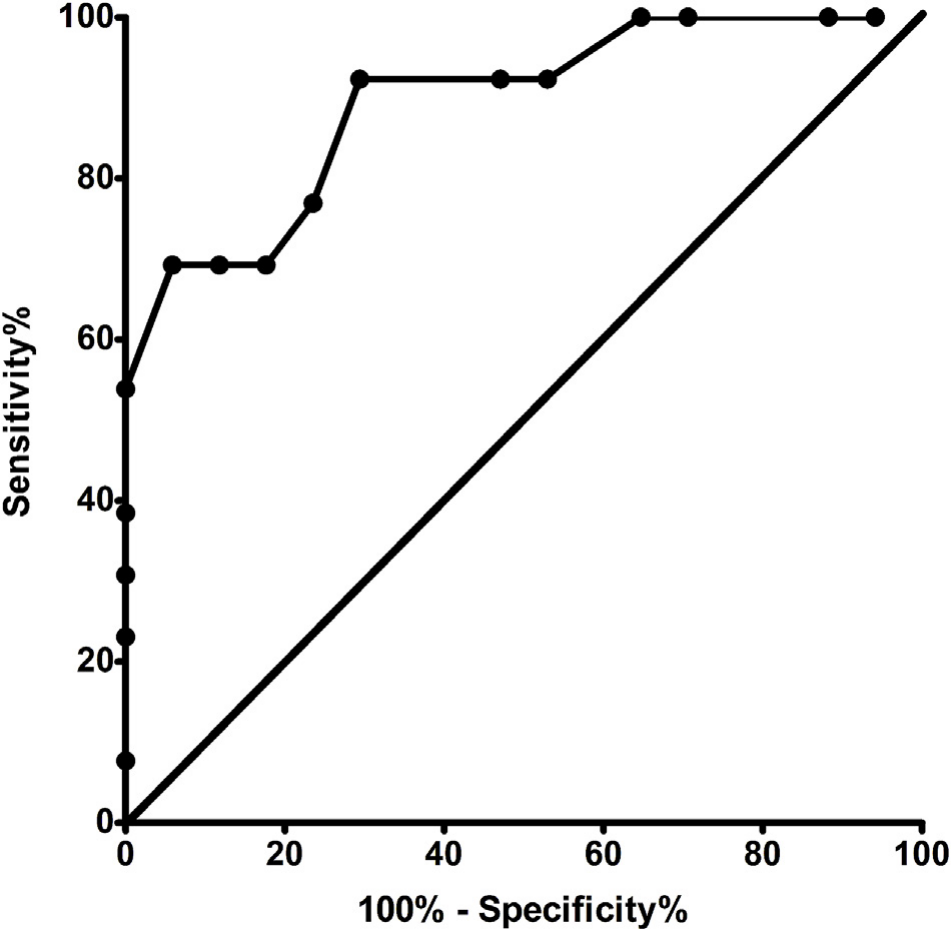
Receiver-Operating characteristic curve for reperfusion hyperkalemia (K > 5.5 mmol/L) by pre reperfusion potassium level. AUC = 0.894 [95%CI, 0.779 to 1.000), p < 0.001. A pre reperfusion potassium level of 4.450 mmol/L was a good predictor of reperfusion hyperkalemia (potassium level over 5.50 mmol/L) (sensitivity: 69.23% (95%CI, 38.57%–90.91%); specificity: 94.12% (95%CI, 71.31%–99.85%; Positive likelihood ratio = 11.77).

**Fig. 3:**
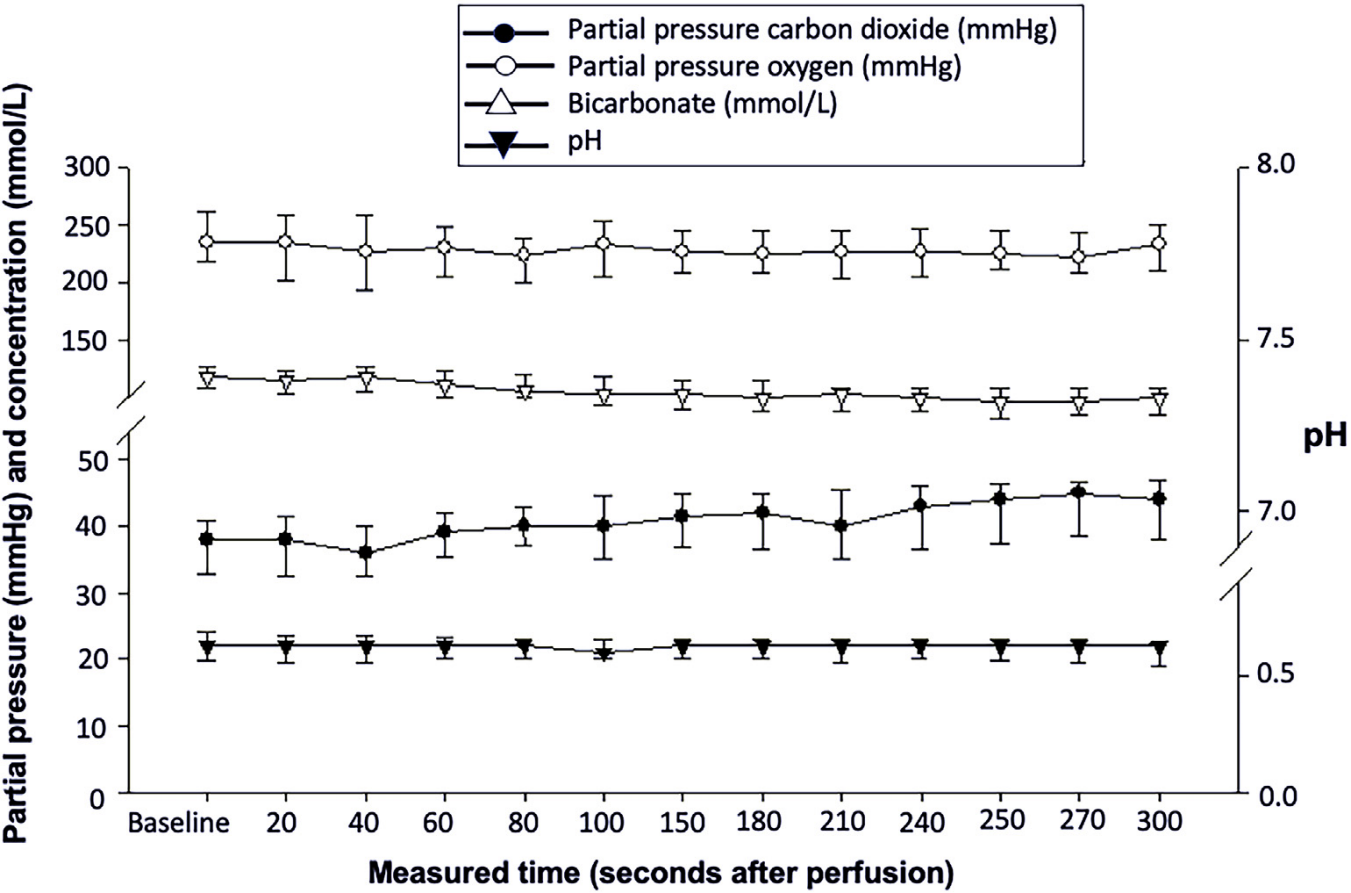
Changes in carbon dioxide, oxygen, pH and bicarbonate during reperfusion. Values are median (interquartile range).

According to the ROC analysis (AUC = 0.894, 95%CI: 0.779 to 1.000, p < 0.001, Fig. 2), a pre reperfusion potassium level of 4.45 mmol/L was a good predictor of reperfusion hyperkalemia (potassium level over 5.50 mmol/L) with a sensitivity of 69.23% (95%CI: 38.57%–90.91%) and a specificity of 94.12% (95%CI: 71.31%–99.85%, positive likelihood ratio = 11.77). Changes in other relevant variables from the blood gas analysis are summarised in Fig. 3.

Regarding patients’ ECG changes during the first 5 min after reperfusion, 7 patients (23%) developed new onset peaked T waves and 3 (10%) patients developed prolongation of PR interval. The one patient who developed life-threatening hyperkalemia with a potassium value of 8 mmol/L at the 40 s time point had widening of their QRS complex. There were no patients who had loss of P waves, “sine wave”, or asystole. Ten patients (33%) had new onset frequent premature ventricular complexes, defined as a run of three or more with a frequency higher than 100 beats/min lasting 30 s or less, without hemodynamic compromise.

All patients received norepinephrine prior to reperfusion with a total median (IQR) dose of 855.0 μg (461.3:1156.0). Nine patients (30%) developed post reperfusion syndrome. Reperfusion syndrome was defined as a decrease of mean arterial pressure and/or heart rate not reaching 30% of baseline value, lasting for less than 5 min, and responsive to an intravenous bolus dose of calcium chloride (1 g) and/or epinephrine (≤100 mcg) without the need to start a second line vasopressor e.g. vasopressin, or escalate the infusion of first line (norepinephrine) vasopressor support by more than 50%.

Twenty-eight patients (93%) were receiving norepinephrine at the time of reperfusion with a median (IQR) dose of 15.0 μg/min (8.2:20.0). The use of vasoactive medications is summarised in Table 2. There was a strong association between pre-reperfusion/baseline potassium levels and peak potassium values during reperfusion (Spearman's Rho = 0.57, 95%CI: 0.26 to 0.77, p = 0.0009). There were no significant correlations observed between changes in peak potassium levels at reperfusion and the following variables - potassium levels during reperfusion, preoperative MELD score, cold and warm ischemia times, potassium effluent levels of the donor liver, norepinephrine dose at the time of reperfusion, total norepinephrine use prior to reperfusion, and length of the anhepatic stage (Table 3).

**Table 2:** Intraoperative fluid and vasoactive medications. Data presented as number (percentile), median (interquartile range).

Variable	Result
**Total operating time (min)**	438.5 (392.5:505.0)
**Surgery stage (min)**	
Dissection (Stage 1)	149.5 (124.0:195.0)
Anhepatic (Stage 2)	69.5 (59.5:77.7)
Neohepatic (Stage 3)	217.0 (164.5:241.0)
**Fluid intervention**	
Plasmalyte solution	
Number of patients	30 (100%)
Total volume (mL)	3500 (2000:5000)
Albumin (20%)	
Number of patients	30 (100%)
Total volume (mL)	600 (400:825)
Red blood cells	
Number of patients	22 (73%)
Total volume (mL)	778.0 (504.8:1507.0)
Platelets	
Number of patients	14 (47%)
Total number of pooled units	4.5 (1.0:5.0)
Fresh frozen plasma	
Number of patients	15 (50%)
Total number of units	2.0 (2.0:4.0)
Cryoprecipitate	
Number of patients	13 (43%)
Total number of units	8.0 (6.0:11.5)
Washed autologous cells	
Number of patients	28 (93%)
Total volume (mL)	911.0 (453.3:1714.0)
Washed donor cells	
Number of patients	10 (33%)
Total volume (mL)	667.0 (446.8:925.0)
**Vasoactive drugs**	
Norepinephrine pre-reperfusion	
Number of patients	30 (100%)
Total dose until reperfusion (μg)	855.0 (461.3:1156.0)
Norepinephrine at reperfusion	
Number of patients	28 (93%)
Dose at reperfusion (μg/min)	15.0 (8.2:20.0)
Epinephrine	
Number of patients	10 (33%)
Total dose (μg)	8.0 (5.0:16.2)
Methylene blue	
Number of patients	3 (10%)
Total dose (mg)	70 (50:100)
Vasopressin	
Number of patients	2 (7%)
Infusion rate (IU/hour)	0.6 (0.2:1.0)
Ephedrine	
Number of patients	1 (3%)
Total dose (mg)	5.0 (3.0:7.0)

**Table 3:** Correlation analysis of factors associated with changes in peak potassium values after liver reperfusion.

Associated factor	Spearman's rho (95% CI)	P value
MELD score	0.02 (−0.39 to 0.35)	0.911
Warm donor liver ischemia time	0.25 (−0.13 to 0.57)	0.169
Cold donor liver ischemia time	−0.064 (−0.31 to 0.42)	0.734
Pre-reperfusion potassium values	0.57 (0.26 to 0.77)	< 0.001
Effluent potassium levels in the donor liver	−0.03 (−0.40 to 0.34)	0.842
Anhepatic time	0.25 (−0.12 to 0.57)	0.169
Total operative time	0.19 (−0.19 to 0.52)	0.307
Total red blood cells	−0.01 (−0.38 to 0.35)	0.939
Norepinephrine dose at reperfusion	0.07 (−0.29 to 0.43)	0.678
Total norepinephrine requirements from start of surgery to reperfusion	−0.25 (−0.57 to 0.12)	0.170

*: p < 0.05 for corresponding correlation coefficient.

### 3.3 Other intraoperative variables and outcomes

The median (IQR) liver dissection time (Stage 1) was 149.5 min (124.0:195.0). Additionally, the median (IQR) anhepatic time (Stage 2) and neohepatic time (Stage 3) were 69.5 min (59.5:77.7) and 217.0 min (164.5:241.0) respectively. All patients received Plasma-Lyte 148 solution and 20% albumin intraoperatively. Total median (IQR) intraoperative Plasma-Lyte 148 solution and 20% albumin use was 3500 mL (2000:2500) and 600 mL (400:825) respectively. Median (IQR) core temperature immediately before and 5 min after reperfusion was 36 °C (35.9:36.7) and 35.7 °C (35.2:35.9), respectively.

Intraoperatively 22 patients (73%) received bank blood with a median (IQR) transfusion volume of 778.0 mL (504.8:1507.0). Fourteen patients (47%) received platelets - median (IQR) 4.5 units (1.0:5.0), 15 patients (50%) received fresh frozen plasma - median (IQR) 2 units (2:4) and 13 patients (43%) received cryoprecipitate - median (IQR) 8.0 units (6.0:11.5). Twenty-eight patients (83%) received washed autologous red blood cells - median (IQR) 911.0 mL (453.3:1714.0) and 10 patients (33%) received washed donor blood with a median (IQR) transfusion volume of 667.0 mL (446.8:925.0). All patients were admitted to the intensive care unit post-transplantation as part of routine hospital protocol. The median (IQR) intensive care unit and total hospital length of stay were 4.5 days (3.0:7.0) and 15.0 days (11.8:38.5) respectively.

## 4 Discussion

### 4.1 Key findings

In adult patients undergoing cadaveric liver transplantation, potassium levels peaked 80 s after reperfusion and lasted 120 s before normalizing to baseline values within 5 min. Reperfusion hyperkalemia was common and occurred in 1 in 5 patients and severe hyperkalemia occurred in 10% of patients with accompanying ECG features. Higher potassium levels immediately prior to reperfusion were associated with higher peak potassium levels after reperfusion. Our findings guide the timing of routine potassium testing during reperfusion and may direct pharmacological and medical prevention and management of hyperkalemia during liver reperfusion.

### 4.2 Relationship to previous findings

The importance of monitoring and managing intraoperative hyperkalemia, particularly during reperfusion of the donor graft has been well recognized. Hyperkalemia has been identified as a major contributor to post-reperfusion cardiac arrest, a finding reported in a large single-centre study that evaluated causes of cardiac arrest in 240 liver transplant recipients [4]. Although abrupt changes in potassium levels are reported to occur after the liver reperfusion phase, only one study has accurately determined the precise time when the potassium values peak after reperfusion [13]. In the study by Acosta et al. [13], 106 patients undergoing liver transplantation were evaluated. The authors reported that potassium levels peaked 30 s after reperfusion. This finding contrasts to the results observed in our study, which demonstrated that potassium values peak 80 s after reperfusion and remained elevated until 120 s post reperfusion. Acosta et al. further reported that by 5 min post-reperfusion, potassium levels had significantly fallen below baseline values, a finding also not reflected in our results; rather we observed that potassium levels returned towards baseline values by 5 min after reperfusion but did not fall below baseline values. The differences in our findings may be multifactorial. Firstly, Acosta et al. may have failed to capture the true peak potassium levels by sampling potassium values only at three timepoints after reperfusion, namely 30 s, 90 s, and 5 min after unclamping of the major vessels. In contrast, we sampled potassium levels every 20 s for the first 2 min after reperfusion, and then every 30 s for a further 3 min. Accordingly, from our 13 time-point samples, we were likely to more accurately determine the peak potassium level after reperfusion. Secondly, in the Acosta et al. study, all patients were pre-emptively treated with atropine, calcium chloride, sodium bicarbonate and hyperventilation prior to reperfusion, interventions not performed in our study.

Other studies have evaluated predictive risk factors for hyperkalemia during living-donor liver transplantation. Xia et al. [14] retrospectively studied 1124 consecutive adult patients who underwent LT and reported that 19.1% of patients were hyperkalemic in the early post-reperfusion period; however, the number of potassium levels sampled was not stated. Similar to our observations, higher baseline potassium levels were associated with post reperfusion hyperkalemia. Juang et al. [15] retrospectively reviewed 487 adult living-donor liver transplantation. Arterial blood gases and electrolytes were samples at least 5 times throughout all stages of surgery; the number of samples post reperfusion was not stated. Compared to our study, 10.4% of patients had hyperkalemia, which was defined as serum potassium levels higher than 5.0 mmol/L. Prolonged anesthesia time, preoperative albumin, and intraoperative blood transfusion were independent risks factors in the development of hyperkalemia. The incidence of post reperfusion hyperkalemia was not reported. Notably, these studies involved live related donors only or a mixed donor cohort, in contrast to our study, which involved only cadaveric donors.

### 4.3 Implications of our findings

**Fig. 4:**
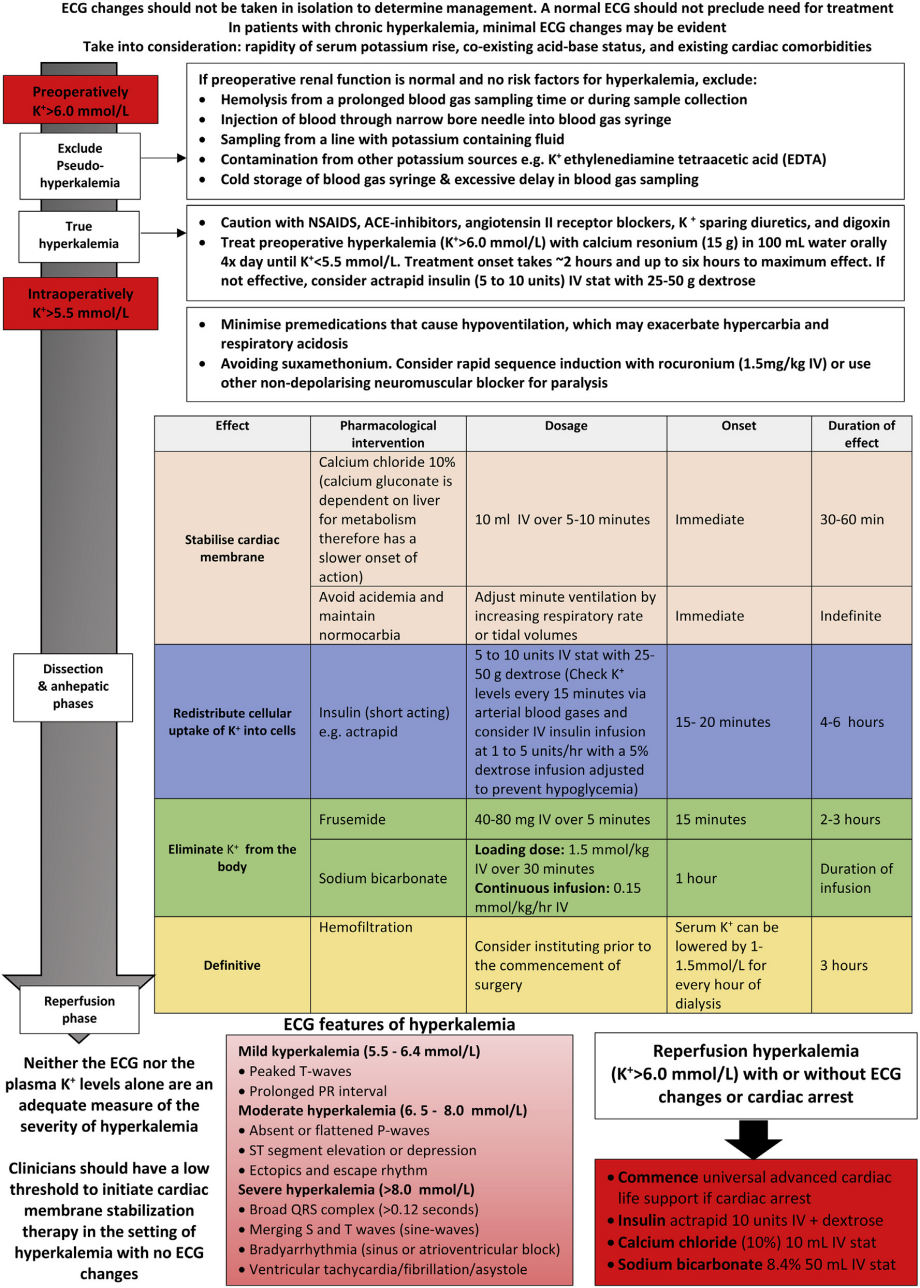
Prevention and management of hyperkalemia during cadaveric liver transplantation.

Anesthetic management of liver transplantation has significantly evolved with advances in medical technology and the establishment of dedicated liver transplant teams [16,17]. However, there are no guidelines regarding timing and frequency of blood sampling during the reperfusion period. The results of our study indicate that potassium levels peak within 2 min post reperfusion and the only modifiable factor associated with post reperfusion hyperkalemia was a higher pre reperfusion/baseline potassium level. This has implications in the pre-emptive prevention of hyperkalemia, the immediate management of potassium levels prior to reperfusion, the postoperative management of hyperkalemia, and any complications that arise from hyperkalemia. Perioperative management strategies for hyperkalemia are summarised in Fig. 4. These include the proactive and aggressive treatment of preoperative hyperkalemia, the rational use of pragmatic intraoperative pharmacological strategies, and the anticipation, recognition and treatment of post reperfusion arrhythmias.

Interestingly we observed that a pre reperfusion potassium level greater than 4.45 mmol/L was strongly predictive of post reperfusion hyperkalemia, suggesting that this may be an appropriate threshold value for pre-emptive treatment in the immediate pre-reperfusion period. From our small cohort, we observed that the sensitivity value from the ROC analysis was moderate (69%). This implies that a potassium level >4.45 mmol/L was able to correctly define reperfusion hyperkalemia in 21 of the 30 patients studied. As such, the development of reperfusion hyperkalemia results not only from a baseline potassium level >4.45 mmol/L, but also from the interplay of other important and contributing factors e.g. reperfusion injury, massive blood transfusion, acute kidney injury, diabetes mellitus etc, all of which need to consider as additional variables in the development of reperfusion hyperkalemia. The high specificity of 94% observed from our ROC analyses further implies that a potassium level <4.45 mmol/L will correctly identify the majority of patients who do not develop reperfusion hyperkalemia. Our findings provide more precise information for optimal timing of blood sampling for potassium and electrolyte monitoring during the reperfusion phase as part of anesthetic liver transplantation protocols. Finally, our findings suggest that hyperkalemia occurs very shortly after reperfusion necessitating extreme vigilance in the early recognition of ECG changes of hyperkalemia at this time point.

### 4.4 Strengths and limitations

There are several strengths and limitations of this study. To our knowledge this is the only study to date to accurately describe the precise pattern and trends of potassium levels during the immediate reperfusion period. Unlike other studies which have only checked for hyperkalemia with one or two blood samples, we have undertaken frequent sampling of blood every 20–30 s. This is a single-centre study of adult patients performed in a high-volume liver transplant centre, partly limiting the external validity of our findings to other institutions. However, our hospital has all the typical characteristics of many tertiary institution liver transplant units, and the surgical and anesthesia transplant protocols adopted are in keeping with those in many other transplant centres. All patients were adults who underwent cadaveric (donation after brain death) liver transplantation, which also limits the generalizability of our findings to donation after cardiac death transplantation, pediatric transplantation, and to adult patients undergoing living related liver transplantation.

Importantly given the exploratory design of this study, we only collected data on 30 consecutive patients. Our findings are hypothesis generating and may provide valuable data for power calculations for future studies on evaluating changes in potassium values during reperfusion. Given the small number of patients, the study is not adequately powered to address any of the secondary outcomes assessed, and larger studies are required to assess more accurately the associations or predictors of hyperkalemia with recipient, donor, intraoperative, and laboratory variables. Given the exploratory nature of the study, we also cannot establish a causal relationship between the perioperative variables we assessed and the impact of these on hyperkalemia, or any other postoperative outcomes. In view of these limitations, we have still provided an in-depth overview of the changes in potassium levels during the reperfusion stage.

Our study has several methodological strengths. The robust sampling of arterial blood gas at 20–30 s intervals allowed us to capture the trend of potassium changes and identify peak values during reperfusion. All acid-base variables were assessed directly from arterial blood and were not amenable to ascertainment bias or derivation. The use of the same blood gas analyzer for all measurements prevented inter-machine analytical bias. Using the same make of blood gas syringes avoided any preanalytical bias in the measurement of potassium concentrations. Further, samples were drawn by a single expert clinician following a standard sampling protocol thereby minimizing any inter-operator bias. Additionally, patients were recruited consecutively and provided with consistent anesthetic management based on current liver transplant protocols. Finally, the blinding of the cardiologist who reported the ECG's to the potassium values minimized any reporting bias.

## 5 Conclusion

Hyperkalemia during cadaveric liver transplantation is common, affecting almost 1 in 2 patients during reperfusion. During reperfusion potassium levels peaked within 2 min and a third of patients developed severe hyperkalemia. Higher potassium levels correlated strongly with higher pre-reperfusion potassium values. Immediately prior to reperfusion of the donor graft, a potassium level of greater than 4.45 mmol/L was a good predictor of reperfusion hyperkalemia. Peak potassium values did not correlate with cold or warm donor ischemia times, duration of the anhepatic stage, norepinephrine use, or effluent potassium levels in the donor liver. These findings guide clinicians with timing of sampling of blood to check for hyperkalemia and in identifying modifiable factors associated with the development of hyperkalemia. Moreover, these findings guide the perioperative management of potassium in patients undergoing cadaveric liver transplantation.

## Authors’ contribution

Laurence Weinberg: conceptualisation; methodology, supervision, investigation, formal analysis, data curation; writing - original draft, review & editing. Dong-Kyu Lee, Kai Wen Leong: formal analysis, data curation; writing - original draft, review & editing. Lachlan Miles, Brett Pearce, Anoop Koshy, Param Pillai: investigation, writing - original draft, review & editing. Ruth Shaylor, Shervin Tosif, Alex Drucker: investigation, review & editing.

## Funding

This research did not receive any specific grant from funding agencies in the public, commercial, or not-for-profit sectors.

## Provenance and peer review

Not commissioned, externally peer reviewed.

## Declaration of competing interest

None.
